# Determination of Coefficients of High-Order Schemes for Riemann-Liouville Derivative

**DOI:** 10.1155/2014/402373

**Published:** 2014-04-15

**Authors:** Rifang Wu, Hengfei Ding, Changpin Li

**Affiliations:** Department of Mathematics, Shanghai University, Shanghai 200444, China

## Abstract

Although there have existed some numerical algorithms for the fractional differential equations, developing high-order methods (i.e., with convergence order greater than or equal to 2) is just the beginning. Lubich has ever proposed the high-order schemes when he studied the fractional linear multistep methods, where he constructed the *p*th order schemes (*p* = 2, 3, 4, 5, 6) for the *α*th order Riemann-Liouville integral and *α*th order Riemann-Liouville derivative. In this paper, we study such a problem and develop recursion formulas to compute these coefficients in the higher-order schemes. The coefficients of higher-order schemes (*p* = 7,8, 9,10) are also obtained. We first find that these coefficients are oscillatory, which is similar to Runge's phenomenon. So, they are not suitable for numerical calculations. Finally, several numerical examples are implemented to testify the efficiency of the numerical schemes for *p* = 3,…, 6.

## 1. Introduction

Loosely speaking, fractional calculus is often regarded as the generalization of classical calculus. From the viewpoint of rigorous mathematics, this is not the case; see [1]. Now, fractional calculus has been successfully applied in the fields of chemistry, physics, finance, signal processing, bioengineering, and control. For details, see [2–11] and references cited therein.

Although there are more than six kinds of fractional derivatives, the commonly used derivatives are Riemann-Liouville, Grünwald-Letnikov, and Caputo ones. In this paper, we focus on Riemann-Liouville derivative. Under suitable conditions, the Riemann-Liouville derivative can be discretized by the discrete form of the Grünwald-Letnikov one.

Some numerical approximate formulas for fractional calculus have been proposed [4, 12–24]. It is worth mentioning that the fractional linear multistep (also high-order) methods for the Riemann-Liouville integrals and derivatives were firstly proposed in [16]. The high-order numerical methods for Caputo derivatives were firstly constructed in [15]. The high-order algorithms for Riesz derivative was firstly considered by Ding et al. [25].

In the following, we give some basic definitions, notations, and properties of the fractional calculus [1, 3, 7, 26].

**Definition 1**

Let *f* be defined on the interval [*a*, *b*] and *α* > 0. Then, the left Riemann-Liouville integral of order *α* is defined as

(1) $$\begin{array}{c} D_{a,x}^{-\alpha }f\left(x\right)=\frac{1}{\Gamma \left(\alpha \right)}\overset{x}{\underset{a}{\int }}\frac{f\left(t\right)}{{\left(x-t\right)}^{1-\alpha }}dt, \end{array}$$

where Γ(·) is Euler's gamma function.

**Definition 2**

Let *f* be defined on the interval (*a*, *b*] and *α* > 0 and let *n* be the smallest integer greater than *α*  (*n* − 1 ≤ *α* < *n*). Then, the left Riemann-Liouville derivative of order *α* is defined by

(2) DRLa,xαf(x)=DnDa,x−(n−α)f(x)=1Γ(n−α)(ddx)n∫axf(t)(x−t)α−n+1dt.

**Definition 3**

Let *f* be defined on the interval [*a*, *b*] and *α* > 0 and let *n* be the smallest integer greater than *α*  (*n* − 1 < *α* ≤ *n*). Then, the left Caputo derivative of order *α* is defined as follows:

(3) DCa,xαf(x)=Da,x−(n−α)Dnf(x)=1Γ(n−α)∫axf(n)(t)(x−t)α−n+1dt.

**Definition 4**

Let *f* be defined on the interval [*a*, *b*] and *α* > 0. Then, the left Grünwald-Letnikov derivative of order *α* is defined as

(4) DGLa,xαf(x)=lim⁡h→0mh=x−a1hα∑l=0m(−1)l(αl)f(x−lh).

**Lemma 5**

Suppose that *f*(*x*) is differentiable in the senses of both Caputo and Riemann-Liouville. Then,

(5) DCa,xαf(x)=DRLa,xαf(x) −∑k=0n−1f(k)(a)Γ(k+1−α)(x−a)k−α,

where *n* − 1 < *α* < *n* ∈ *Z*_+_.

**Lemma 6**

Let *f* ∈ *C* ^*n*^[*a*, *b*]; then the finite Grünwald-Letnikov derivative

(6) D~GLa,xαf(x)=1hα∑l=0[(x−a)/h](−1)l(αl)f(x−lh)

yields a first-order approximation for the Riemann-Liouville derivative _*RL*_ *D*_*a*,*x*_^*α*^ *f*(*x*) if *f*(*a*+) = 0; that is,

(7) DRLa,xαf(x)=D~GLa,xαf(x)+O(h).

But when *f*(*a*+) ≠ 0, then one has

(8) DRLa,xαf(x)=D~GLa,xαf(x)+O(h)+O(f(a+)).

The present paper is organized as follows. We divide Section 2 into three subsections. In Section 2.1, we introduce the recursion formulas of coefficients of orders 1 and 2, which are usually used, and some properties are given. In Section 2.2, coefficients of orders from 3 to 6 are presented for reference. And coefficients of orders from 7 to 10 are displayed in Section 2.3 which are oscillatory. Such a phenomenon is similar to Runge's phenomenon. So, the high-order (more than 6th-order) schemes seem not to be suitable for numerical calculations, which is the same as the case of ordinary differential equation. In Section 3, some numerical experiments are carried out to support the computational schemes.  Section 4 concludes this paper.

## 2. The Determination of the Coefficients

Firstly, we divide the given interval [*a*, *b*] into

(9) $$\begin{array}{c} \Lambda :a=x_{0}<x_{1}<\cdots <x_{M}=b \end{array}$$

and *x*_*m*_ = *a* + *mh*, in which *h* = (*b* − *a*)/*M*, *m* = 0,1,…, *M*.

In most situations, we naturally use the following formula to approximate the Riemann-Liouville derivative:

(10) DRLa,xαf(xm)=1hα∑l=0mϖ1,l(α)f(xm−l)+O(h)+O(f(a+)),m=0,1,…,M,

where

(11) ϖ1,l(α)=(−1)l(αl)=(−1)lΓ(α+1)Γ(l+1)Γ(α−l+1),l=0,1,…,m,

are the binomial coefficients. And they have the following recurrence relationships:

(12) ϖ1,0(α)=1,ϖ1,l(α)=(1−1+αl)ϖ1,l−1(α), l=1,2,…,m.

Obviously, *ϖ*_1,*l*_^(*α*)^  (*l* = 0,1,…, *m*) are just the first *m* + 1 coefficients of Taylor series of the expansion of the following function:

(13) $$\begin{array}{c} W_{1}^{\left(\alpha \right)}\left(z\right)={\left(1-z\right)}^{\alpha }=\overset{\infty }{\underset{l=0}{\sum }}{\left(-1\right)}^{l}\left(\begin{array}{c} \alpha \\ l \end{array}\right)z^{l}=\overset{\infty }{\underset{l=0}{\sum }}\varpi_{1,l}^{\left(\alpha \right)}z^{l}. \end{array}$$

We can see that formula (10) has only the first-order accuracy if *f*(*a*+) = 0. Therefore, to seek high accurate numerical methods for the fractional derivatives is of great importance. In [16], Lubich firstly proposed numerical schemes of orders 2, 3, 4, 5, and 6 Riemann-Liouvile derivative, named fractional linear multistep formulas. Here, it must be mentioned that the fractional linear multistep method is different from the usual linear multistep method. The former is of varied steps. That is to say, the value of the *m*th step *x*_*m*_ relies on the preceding step values *x*_0_, *x*_1_,…, *x*_*m*−1_, which means that the number of multisteps is increasing, while the latter is of fixed number of multisteps. Under the homogeneous initial value conditions, that is, *f* ^(*k*)^(*a*+) = 0  (*k* = 0,1,…, *p* − 1), the fractional linear multistep scheme for the Riemann-Liouville derivative has the following form:

(14) DRLa,xαf(xm)=1hα∑l=0mϖp,l(α)f(xm−l)+O(hp),p=2,…,10.

If *p* ≥ 2, the corresponding numerical methods are often called the high-order methods. The coefficients *ϖ*_*p*,*l*_^(*α*)^ in the above equations are those of the Taylor series expansions of the corresponding generating functions *W*_*p*_^(*α*)^(*z*); that is,

(15) $$\begin{array}{c} W_{p}^{\left(\alpha \right)}\left(z\right)=\overset{\infty }{\underset{l=0}{\sum }}\varpi_{p,l}^{\left(\alpha \right)}z^{l},\;p=2,\ldots ,10. \end{array}$$

The corresponding generating functions for *p* = 2,…, 6 are given as follows [16]:

(16) W2(α)(z)=(32−2z+12z2)α,W3(α)(z)=(116−3z+32z2−13z3)α,W4(α)(z)=(2512−4z+3z2−43z3+14z4)α,W5(α)(z)=(13760−5z+5z2−103z3+54z4−15z5)α,W6(α)(z)=(14760−6z+152z2−203z3+154z4−65z5+16z6)α.

Using the similar method, we list the generating functions for *p* = 7,…, 10,

(17) W7(α)(z)=(363140−7z+212z2−353z3+354z4−215z5+76z6−17z7)α,W8(α)(z)=(761280−8z+14z2−563z3+352z4−565z5+143z6−87z7+18z8)α,W9(α)(z)=(71292520−9z+18z2−28z3+632z4−1265z5+14z6−367z7+98z8−19z9)α,W10(α)(z)=(147625040−10z+452z2−40z3+1052z4−2525z5+35z6−1207z7+458z8−109z9+110z10)α.

From the above introduction, the key question is how to compute the coefficients *ϖ*_*p*,*l*_^(*α*)^, *p* = 2,…, 10. As far as we know, there are three methods to compute these coefficients, in which one way is to use the fast Fourier transform method [19].

Another way of computing the coefficients *ϖ*_*p*,*l*_^(*α*)^, *p* = 2,…, 10, is by the automatic differentiation techniques [13]

(18) $$\begin{array}{c} \varpi_{p,l}^{\left(\alpha \right)}=\frac{1}{lu_{0}}\overset{l-1}{\underset{j=0}{\sum }}\left[\alpha \left(l-j\right)-j\right]\varpi_{p,j}^{\left(\alpha \right)}u_{l-j},\;p=2,\ldots ,10. \end{array}$$

Here, the values *u*_*k*_  (*k* = 0,1,…) in the above formula denote the Taylor expansion coefficients of the generating functions *W*_*p*_^(*α*)^(*z*)  (*p* = 2,…, 10) of the classical linear multistep methods.

The third method is to use the expansion of series, where the coefficients are not given in the form of recurrence relationships but the exact expressions; see the Appendix. Such analytical expressions are useful for theoretical analysis, such as stability and convergence. Here, we introduce the second method.

### 2.1. Properties of *ϖ*_1,*l*_^(*α*)^ and *ϖ*_2,*l*_^(*α*)^

As far as we know, the coefficients of the 1st-order and 2nd-order schemes are often used. Here, we present their properties for reference. We know that *ϖ*_1,*l*_^(*α*)^ are the binomial coefficients in formula (12). They have the following properties.

**Property 1**

The coefficients *ϖ*_1,*l*_^(*α*)^  (*l* = 0,1,…) for 0 < *α* < 1 have the following properties:

1. *ϖ*_1,0_^(*α*)^ = 1, *ϖ*_1,*l*_^(*α*)^ = (−1)^*l*^Γ(*α* + 1)/(Γ(*l* + 1)Γ(*α* − *l* + 1)) < 0, *l* ≥ 1;
2. *ϖ*_1,*l*−1_^(*α*)^ ≤ *ϖ*_1,*l*_^(*α*)^, *l* ≥ 2;
3. ∑_*l*=0_^*∞*^ *ϖ*_1,*l*_^(*α*)^ = 0, lim⁡_*l*→*∞*_⁡*ϖ*_1,*l*_^(*α*)^ = 0.

Parallelly, the properties of the coefficients of the 1st-order schemes for 1 < *α* < 2 are given as follows.

*Property* *1*′. The coefficients *ϖ*_1,*l*_^(*α*)^  (*l* = 0,1,…) for 1 < *α* < 2 have the following properties:

1. *ϖ*_1,0_^(*α*)^ = 1, *ϖ*_1,1_ = −*α* < 0, *ϖ*_1,*l*_^(*α*)^ = (−1)^*l*^Γ(*α* + 1)/(Γ(*l* + 1)Γ(*α* − *l* + 1)) > 0, *l* ≥ 2;
2. *ϖ*_1,*l*−1_^(*α*)^ ≥ *ϖ*_1,*l*_^(*α*)^, *l* ≥ 3;
3. ∑_*l*=0_^*∞*^ *ϖ*_1,*l*_^(*α*)^ = 0, lim⁡_*l*→*∞*_⁡*ϖ*_1,*l*_^(*α*)^ = 0.

The above Properties 1 and 1′ are known to us. Next, we present the coefficients *ϖ*_2,*l*_^(*α*)^ which are somewhat complex. Let *i* = *l* − *j*; formula (18) can be written as

(19) $$\begin{array}{c} \varpi_{p,l}^{\left(\alpha \right)}=\frac{1}{lu_{0}}\overset{l}{\underset{i=1}{\sum }}\left(\alpha i-l+i\right)\varpi_{p,l-i}^{\left(\alpha \right)}u_{i},\;p=2,\ldots ,10. \end{array}$$

In case *p* = 2, the corresponding generating function is *W*_2_^(*α*)^(*z*). So, *u*_0_ = 3/2, *u*_1_ = −2, *u*_2_ = 1/2, *u*_*i*_ = 0, *i* = 3,4,…, and *ϖ*_2,*l*_^(*α*)^ are coefficients of the Taylor series expansion of  *W*_2_^(*α*)^(*z*); it is easy to get

(20) $$\begin{array}{c} \varpi_{2,0}^{\left(\alpha \right)}={\left(\frac{3}{2}\right)}^{\alpha }. \end{array}$$

From (19), we have

(21) ϖ2,1(α)=1u0(α−1+1)ϖ2,0(α)u1=−43αϖ2,0(α),ϖ2,l(α)=1lu0[(α−l+1)ϖ2,l−1(α)u1+(2α−l+2)ϖ2,l−2(α)u2]=23l[−2(α−l+1)ϖ2,l−1(α)+12(2α−l+2)ϖ2,l−2(α)],l=3,4,….

By the tedious calculations, one has the following result.

**Property 2**

The coefficients *ϖ*_2,*l*_^(*α*)^  (*l* = 0,1,…) for 0 < *α* < 1 have the following properties:

(1) *ϖ*_2,0_^(*α*)^ = (3/2)^*α*^ > 0, *ϖ*_2,1_^(*α*)^ = −(4/3)*αϖ*_2,0_^(*α*)^ < 0;
  (22) ϖ2,2(α)=α(8α−5)9(32)α≤0 for  0<α≤58,ϖ2,2(α)=α(8α−5)9(32)α>0 for  58<α<1;ϖ2,3(α)=4α(1−α)(8α−7)81(32)α≤0 for  0<α≤78,ϖ2,3(α)=4α(1−α)(8α−7)81(32)α>0 for  78<α<1;ϖ2,l(α)=23l[−2(α−l+1)ϖ2,l−1(α)+12(2α−l+2)ϖ2,l−2(α)]<0, l=4,5,…
(2) *ϖ*_2,*l*−1_^(*α*)^ ≤ *ϖ*_2,*l*_^(*α*)^, *l* ≥ 5;
(3) ∑_*l*=0_^*∞*^ *ϖ*_2,*l*_^(*α*)^ = 0, lim⁡_*l*→*∞*_⁡*ϖ*_2,*l*_^(*α*)^ = 0.

Similarly, the coefficients of the 2nd scheme for 1 < *α* < 2 have the following properties.

*Property* *2*′. The coefficients *ϖ*_2,*l*_^(*α*)^  (*l* = 0,1,…) for 1 < *α* < 2 have the following properties;

(1) *ϖ*_2,0_^(*α*)^ = (3/2)^*α*^ > 0, *ϖ*_2,1_^(*α*)^ = −(4/3)*αϖ*_2,0_^(*α*)^ < 0;
  (23) ϖ2,2(α)=α(8α−5)9(32)α>0;ϖ2,3(α)=4α(1−α)(8α−7)81(32)α<0;ϖ2,l(α)=23l[−2(α−l+1)ϖ2,l−1(α)+12(2α−l+2)ϖ2,l−2(α)]>0, l=4,5,…
(2) *ϖ*_2,*l*−1_^(*α*)^ ≥ *ϖ*_2,*l*_^(*α*)^, *l* ≥ 5;
(3) ∑_*l*=0_^*∞*^ *ϖ*_2,*l*_^(*α*)^ = 0, lim⁡_*l*→*∞*_⁡*ϖ*_2,*l*_^(*α*)^ = 0.

The proofs of Properties 2 and 2′ can refer to [4].

### 2.2. Determination of *ϖ*_*p*,*l*_^(*α*)^, *p*  =  3,4, 5,6

In this subsection, we present the recurrence relationships of the coefficients *ϖ*_*p*,*l*_^(*α*)^  (*p* = 3,4, 5,6) for reference.

(1) When *p* = 3, then *u*_0_ = 11/6, *u*_1_ = −3, *u*_2_ = 3/2, *u*_3_ = −1/3, *u*_*i*_ = 0, *i* = 4,5,…,

(24) ϖ3,0(α)=(116)α,ϖ3,1(α)=1u0(α−1+1)ϖ3,0(α)u1=−1811αϖ3,0(α),ϖ3,2(α)=12u0[(α−2+1)ϖ3,1(α)u1+(2α−2+2)ϖ3,0(α)u2]=311[−3(α−1)ϖ3,1(α)+3αϖ3,0(α)],ϖ3,l(α)=1lu0[(α−l+1)ϖ3,l−1(α)u1+(2α−l+2)ϖ3,l−2(α)u2    +(3α−l+3)ϖ3,l−3(α)u3]=611l[−3(α−l+1)ϖ3,l−1(α)+32(2α−l+2)ϖ3,l−2(α)−13(3α−l+3)ϖ3,l−3(α)], l=3,4,….

(2) When *p* = 4, then *u*_0_ = 25/12, *u*_1_ = −4, *u*_2_ = 3, *u*_3_ = −4/3, *u*_4_ = 1/4, *u*_*i*_ = 0, *i* = 5,6,…,

(25) ϖ4,0(α)=(2512)α,ϖ4,1(α)=1u0(α−1+1)ϖ4,0(α)u1=−4825αϖ4,0(α),ϖ4,2(α)=625[−4(α−1)ϖ4,1(α)+6αϖ4,0(α)],ϖ4,3(α)=425[−4(α−2)ϖ4,2(α)+3(2α−1)ϖ4,1(α)−4αϖ4,0(α)],ϖ4,l(α)=1225l[−4(α−l+1)ϖ4,l−1(α)+3(2α−l+2)ϖ4,l−2(α)−43(3α−l+3)ϖ4,l−3(α)+14(4α−l+4)ϖ4,l−4(α)], l=4,5,….

(3) When *p* = 5, then *u*_0_ = 137/60, *u*_1_ = −5, *u*_2_ = 5, *u*_3_ = −10/3, *u*_4_ = 5/4, *u*_5_ = −1/5, *u*_*i*_ = 0, *i* = 6,7,…,

(26) ϖ5,0(α)=(13760)α,ϖ5,1(α)=−300137αϖ5,0(α),ϖ5,2(α)=30137[−5(α−1)ϖ5,1(α)+10αϖ5,0(α)],ϖ5,3(α)=20137[−5(α−2)ϖ5,2(α)+5(2α−1)ϖ5,1(α)−10αϖ5,0(α)],ϖ5,4(α)=15137[−5(α−3)ϖ5,3(α)+5(2α−2)ϖ5,2(α)−103(3α−1)ϖ5,1(α)+5αϖ5,0(α)],ϖ5,l(α)=60137l[−5(α−l+1)ϖ5,l−1(α)+5(2α−l+2)ϖ5,l−2(α)−103(3α−l+3)ϖ5,l−3(α)+54(4α−l+4)ϖ5,l−4(α)−15(5α−l+5)ϖ5,l−5(α)], l=5,6,….

(4) When *p* = 6, then *u*_0_ = 147/60, *u*_1_ = −6, *u*_2_ = 15/2, *u*_3_ = −20/3, *u*_4_ = 15/4, *u*_5_ = −6/5, *u*_6_ = 1/6, *u*_*i*_ = 0, *i* = 7,8,…,

(27) ϖ6,0(α)=(14760)α,ϖ6,1(α)=−360147αϖ6,0(α),ϖ6,2(α)=30147[−6(α−1)ϖ6,1(α)+15αϖ6,0(α)],ϖ6,3(α)=20147[−6(α−2)ϖ6,2(α)+152(2α−1)ϖ6,1(α)−20αϖ6,0(α)],ϖ6,4(α)=15147[−6(α−3)ϖ6,3(α)+152(2α−2)ϖ6,2(α)−203(3α−1)ϖ6,1(α)+15αϖ6,0(α)],ϖ6,5(α)=12147[−6(α−4)ϖ6,4(α)+152(2α−3)ϖ6,3(α)−203(3α−2)ϖ6,2(α)+154(4α−1)ϖ6,1(α)−6αϖ6,0(α)],ϖ6,l(α)=60147l[−6(α−l+1)ϖ6,l−1(α)+152(2α−l+2)ϖ6,l−2(α)−203(3α−l+3)ϖ6,l−3(α)+154(4α−l+4)ϖ6,l−4−65(5α−l+5)ϖ6,l−5(α)+16(6α−l+6)ϖ6,l−6], l=6,7,….

**Figure 1:**
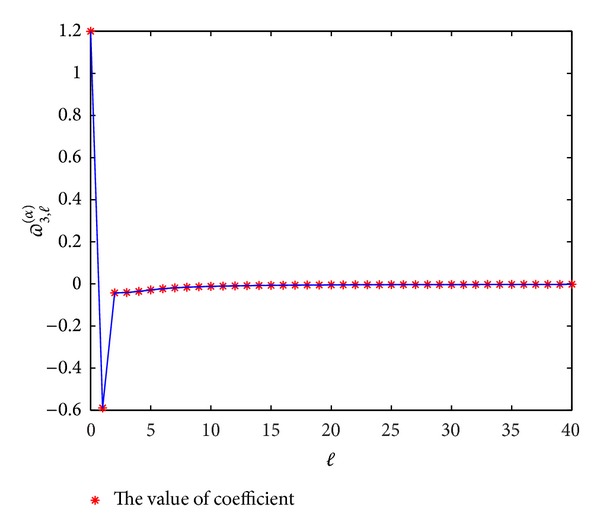
The values of the coefficients *ϖ*_3,*l*_^(*α*)^  (*l* = 0,1,…) for *α* = 0.3.

**Figure 2:**
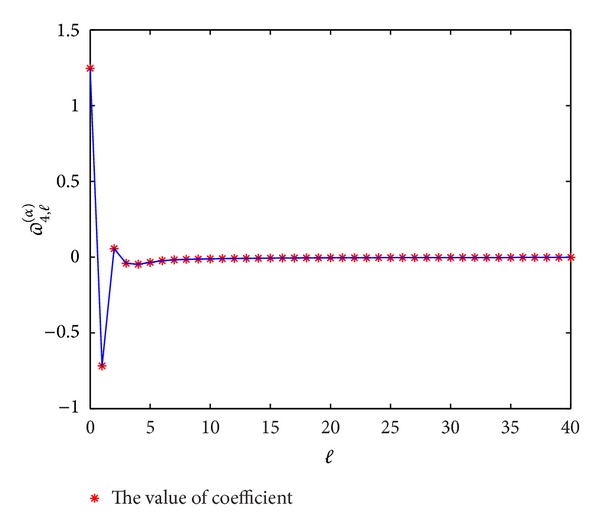
The values of the coefficients *ϖ*_4,*l*_^(*α*)^  (*l* = 0,1,…) for *α* = 0.3.

**Figure 3:**
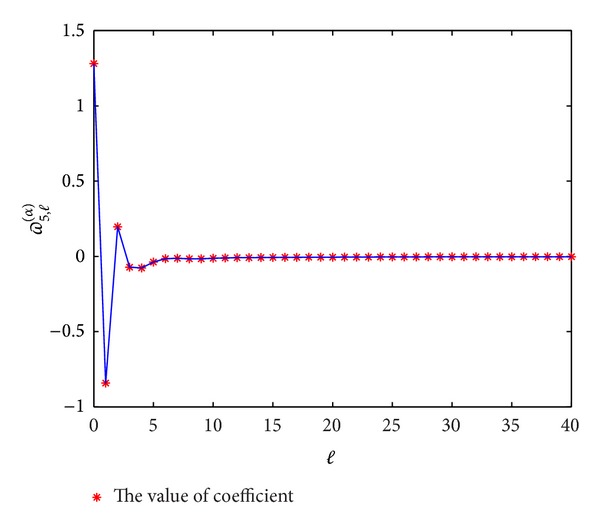
The values of the coefficients *ϖ*_5,*l*_^(*α*)^  (*l* = 0,1,…) for *α* = 0.3.

**Figure 4:**
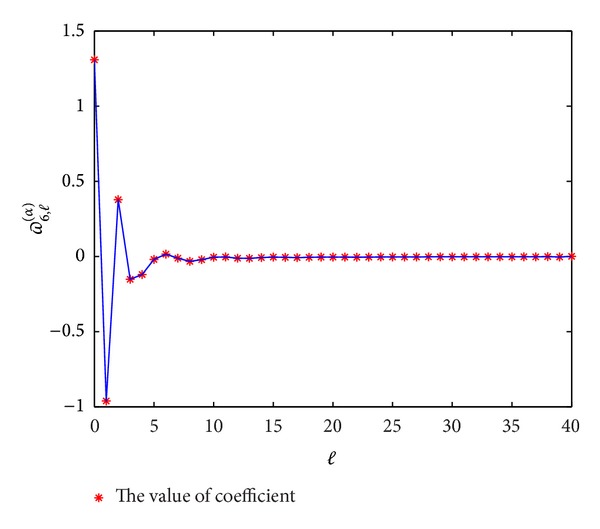
The values of the coefficients *ϖ*_6,*l*_^(*α*)^  (*l* = 0,1,…) for *α* = 0.3.

In Figures 1, 2, 3, and 4, we display the coefficients *ϖ*_*p*,*l*_^(*α*)^  (*p* = 3,4, 5,6) for different *α*; it can be seen that *ϖ*_*p*,*l*_^(*α*)^ → 0 when *l* → *∞*, which coincides with the convergence [16].

**Remark 7**

In [15], the high-order schemes for Caputo derivative were firstly derived. Here, one can get another way to construct the high-order numerical algorithms for Caputo derivatives. If the homogeneous initial value conditions are satisfied, one has the following numerical schemes due to Lemma 5:

(28) DCa,xαf(xm)=1hα∑l=0mϖp,l(α)f(xm−l)+O(hp),p=2,3,4,5,6.

### 2.3. Determination of *ϖ*_*p*,*l*_  (*p*  =  7,8, 9,10)

In this subsection, we present the recursion formulas of *ϖ*_*p*,*l*_  (*p* = 7,8, 9,10) for reference.

(1) When *p* = 7, then *u*_0_ = 363/140, *u*_1_ = −7, *u*_2_ = 21/2, *u*_3_ = −35/3, *u*_4_ = 35/4, *u*_5_ = −21/5, *u*_6_ = 7/6, *u*_7_ = −1/7, and *u*_*i*_ = 0, *i* = 8,9,…. The coefficients are given as follows:

(29) ϖ7,0(α)=(363140)α,ϖ7,1(α)=−980363αϖ7,0(α),ϖ7,2(α)=70363[−7(α−1)ϖ7,1(α)+21αϖ7,0(α)],ϖ7,3(α)=1401089[−7(α−2)ϖ7,2(α)+212(2α−1)ϖ7,1(α)−35αϖ7,0(α)],ϖ7,4(α)=35363[−7(α−3)ϖ7,3(α)+21(α−1)ϖ7,2(α)−353(3α−1)ϖ7,1(α)+35αϖ7,0(α)],ϖ7,5(α)=28363[−7(α−4)ϖ7,4(α)+212(2α−3)ϖ7,3(α)−353(3α−2)ϖ7,2(α)+354(4α−1)ϖ7,1(α)−21αϖ7,0(α)],ϖ7,6(α)=701089[−7(α−5)ϖ7,5(α)+21(α−2)ϖ7,4(α)−35(α−1)ϖ7,3(α)+352(2α−1)ϖ7,2(α)−215(5α−1)ϖ7,1(α)+7αϖ7,0(α)],ϖ7,l=140363l[−7(α−l+1)ϖ7,l−1(α)+212(2α−l+2)ϖ7,l−2(α)−353(3α−l+3)ϖ7,l−3(α)+354(4α−l+4)ϖ7,l−4(α)−215(5α−l+5)ϖ7,l−5(α)+76(6α−l+6)ϖ7,l−6(α)−17(7α−l+7)ϖ7,l−7(α)], l=7,8,….

(2) When *p* = 8, then *u*_0_ = 761/280, *u*_1_ = −8, *u*_2_ = 14, *u*_3_ = −56/3, *u*_4_ = 35/2, *u*_5_ = −56/5, *u*_6_ = 14/3, *u*_7_ = −8/7, *u*_8_ = 1/8, and *u*_*i*_ = 0, *i* = 9,10,…. The coefficients are given as follows:

(30) ϖ8,0(α)=(761280)α,ϖ8,1(α)=−2240761αϖ8,0(α),ϖ8,2(α)=140761[−8(α−1)ϖ8,1(α)+28αϖ8,0(α)],ϖ8,3(α)=2802283[−8(α−2)ϖ8,2(α)+14(2α−1)ϖ8,1(α)−56αϖ8,0(α)],ϖ8,4(α)=70761[−8(α−3)ϖ8,3(α)+28(α−1)ϖ8,2(α)−563(3α−1)ϖ8,1(α)+70αϖ8,0(α)],ϖ8,5(α)=56761[−8(α−4)ϖ8,4(α)+14(2α−3)ϖ8,3(α)−563(3α−2)ϖ8,2(α)+352(4α−1)ϖ8,1(α)−56αϖ8,0(α)],ϖ8,6(α)=1402283[−8(α−5)ϖ8,5(α)+28(α−2)ϖ8,4(α)−56(α−1)ϖ8,3(α)+35(2α−1)ϖ8,2(α)−565(5α−1)ϖ8,1(α)+28αϖ8,0(α)],ϖ8,7(α)=40761[−8(α−6)ϖ8,6(α)+14(2α−5)ϖ8,5(α)−563(3α−4)ϖ8,4(α)+352(4α−3)ϖ8,3(α)−565(5α−2)ϖ8,2(α)+143(6α−1)ϖ8,1(α)−8αϖ8,0(α)],ϖ8,l(α)=280761l[−8(α−l+1)ϖ8,l−1(α)+14(2α−l+2)ϖ8,l−2(α)−563(3α−l+3)ϖ8,l−3(α)+352(4α−l+4)ϖ8,l−4(α)−565(5α−l+5)ϖ8,l−5(α)+143(6α−l+6)ϖ8,l−6(α)−87(7α−l+7)ϖ8,l−7(α)+18(8α−l+8)ϖ8,l−8(α)], l=8,9,….

(3) When *p* = 9, then *u*_0_ = 7129/2520, *u*_1_ = −9, *u*_2_ = 18, *u*_3_ = −28, *u*_4_ = 63/2, *u*_5_ = −126/5, *u*_6_ = 14, *u*_7_ = −36/7, *u*_8_ = 9/8, and *u*_9_ = −1/9, *u*_*i*_ = 0, *i* = 10,11,…. The coefficients are displayed as follows:

(31) ϖ9,0(α)=(71292520)α,ϖ9,1(α)=−226807129αϖ9,0(α),ϖ9,2(α)=12607129[−9(α−1)ϖ9,1(α)+36αϖ9,0(α)],ϖ9,3(α)=8407129[−9(α−2)ϖ9,2(α)+18(2α−1)ϖ9,1(α)−84αϖ9,0(α)],ϖ9,4(α)=6307129[−9(α−3)ϖ9,3(α)+36(α−1)ϖ9,2(α)−28(3α−1)ϖ9,1(α)+126αϖ9,0(α)],ϖ9,5(α)=5047129[−9(α−4)ϖ9,4(α)+18(2α−3)ϖ9,3(α)−28(3α−2)ϖ9,2(α)+632(4α−1)ϖ9,1(α)−126αϖ9,0(α)],ϖ9,6(α)=4207129[−9(α−5)ϖ9,5(α)+36(α−2)ϖ9,4(α)−84(α−1)ϖ9,3(α)+63(2α−1)ϖ9,2(α)−1265(5α−1)ϖ9,1(α)+84αϖ9,0(α)],ϖ9,7(α)=3607129[−9(α−6)ϖ9,6(α)+18(2α−5)ϖ9,5(α)−28(3α−4)ϖ9,4(α)+632(4α−3)ϖ9,3(α)−1265(5α−2)ϖ9,2(α)+14(6α−1)ϖ9,1(α)−36αϖ9,0(α)],ϖ9,8(α)=3157129[−9(α−7)ϖ9,7(α)+36(α−3)ϖ9,6(α)−28(3α−5)ϖ9,5(α)+126(α−1)ϖ9,4(α)−1265(5α−3)ϖ9,3(α)+28(3α−1)ϖ9,2(α)−367(7α−1)ϖ9,1(α)+72αϖ9,0(α)],ϖ9,l(α)=25207129l[−9(α−l+1)ϖ9,l−1(α)+18(2α−l+2)ϖ9,l−2(α)−28(3α−l+3)ϖ9,l−3(α)+632(4α−l+4)ϖ9,l−4(α)−1265(5α−l+5)ϖ9,l−5(α)+14(6α−l+6)ϖ9,l−6(α)−367(7α−l+7)ϖ9,l−7(α)+98(8α−l+8)ϖ9,l−8(α)−19(9α−l+9)ϖ9,l−9(α)], l=9,10,….

(4) When *p* = 10, then *u*_0_ = 7381/2520, *u*_1_ = −10, *u*_2_ = 45/2, *u*_3_ = −40, *u*_4_ = 105/2, *u*_5_ = −252/5, *u*_6_ = 35, *u*_7_ = −120/7, *u*_8_ = 45/8, *u*_9_ = −10/9, *u*_10_ = 1/10, and *u*_*i*_ = 0, *i* = 11,12,…. The coefficients are given as follows:

(32) ϖ10,0(α)=(73812520)α,ϖ10,1(α)=−252007381αϖ10,0(α),ϖ10,2(α)=12607381[−10(α−1)ϖ10,1(α)+45αϖ10,0(α)],ϖ10,3(α)=8407381[−10(α−2)ϖ10,2(α)+452(2α−1)ϖ10,1(α)−120αϖ10,0(α)],ϖ10,4(α)=6307381[−10(α−3)ϖ10,3(α)+45(α−1)ϖ10,2(α)−40(3α−1)ϖ10,1(α)+210αϖ10,0(α)],ϖ10,5(α)=5047381[−10(α−4)ϖ10,4(α)+452(2α−3)ϖ10,3(α)−40(3α−2)ϖ10,2(α)+1052(4α−1)ϖ10,1(α)−252αϖ10,0(α)],ϖ10,6(α)=4207381[−10(α−5)ϖ10,5(α)+45(α−2)ϖ10,4(α)−120(α−1)ϖ10,3(α)+105(2α−1)ϖ10,2(α)−2525(5α−1)ϖ10,1(α)+210αϖ10,0(α)],ϖ10,7(α)=3607381[−10(α−6)ϖ10,6(α)+452(2α−5)ϖ10,5(α)−40(3α−4)ϖ10,4(α)+1052(4α−3)ϖ10,3(α)−2525(5α−2)ϖ10,2(α)+35(6α−1)ϖ10,1(α)−120αϖ10,0(α)],ϖ10,8(α)=3157381[−10(α−7)ϖ10,7(α)+45(α−3)ϖ10,6(α)−40(3α−5)ϖ10,5(α)+210(α−1)ϖ10,4(α)−2525(5α−3)ϖ10,3(α)+70(3α−1)ϖ10,2(α)−1207(7α−1)ϖ10,1(α)+45αϖ10,0(α)],ϖ10,9(α)=2807381[−10(α−8)ϖ10,8(α)+452(2α−7)ϖ10,7(α)−120(α−2)ϖ10,6(α)+1052(4α−5)ϖ10,5(α)−2525(5α−4)ϖ10,4(α)+105(2α−1)ϖ10,3(α)−1207(7α−2)ϖ10,2(α)+458(8α−1)ϖ10,1(α)−10αϖ10,0(α)],ϖ10,l(α)=25207381l[−10(α−l+1)ϖ10,l−1(α)+452(2α−l+2)ϖ10,l−2(α)−40(3α−l+3)ϖ10,l−3(α)+1052(4α−l+4)ϖ10,l−4(α)−2525(5α−l+5)ϖ10,l−5(α)+35(6α−l+6)ϖ10,l−6(α)−1207(7α−l+7)ϖ10,l−7(α)+458(8α−l+8)ϖ10,l−8(α)−109(9α−l+9)ϖ10,l−9(α)+110(10α−l+10)ϖ10,10−l(α)],l=10,11,….

**Figure 5:**
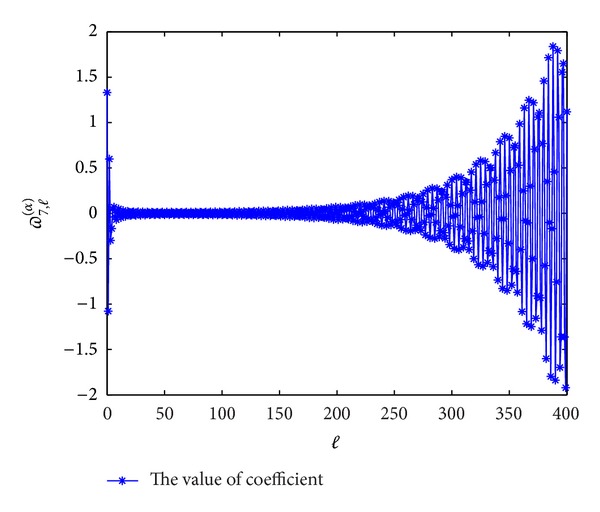
The values of the coefficients *ϖ*_7,*l*_^(*α*)^  (*l* = 0,1,…) for *α* = 0.3.

**Figure 6:**
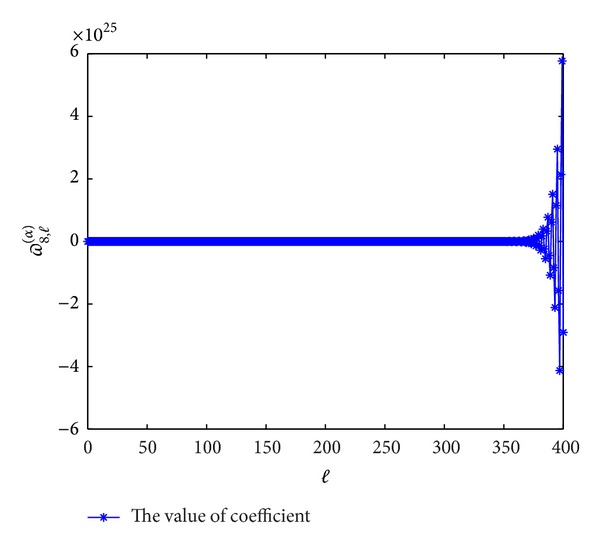
The values of the coefficients *ϖ*_8,*l*_^(*α*)^  (*l* = 0,1,…) for *α* = 0.3.

**Figure 7:**
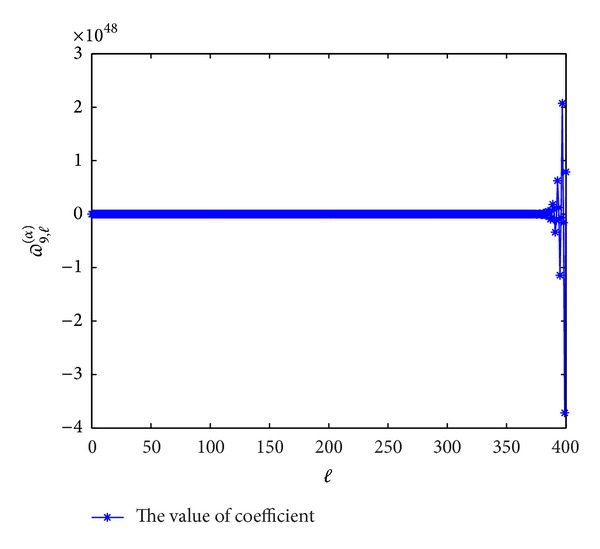
The values of the coefficients *ϖ*_9,*l*_^(*α*)^  (*l* = 0,1,…) for *α* = 0.3.

**Figure 8:**
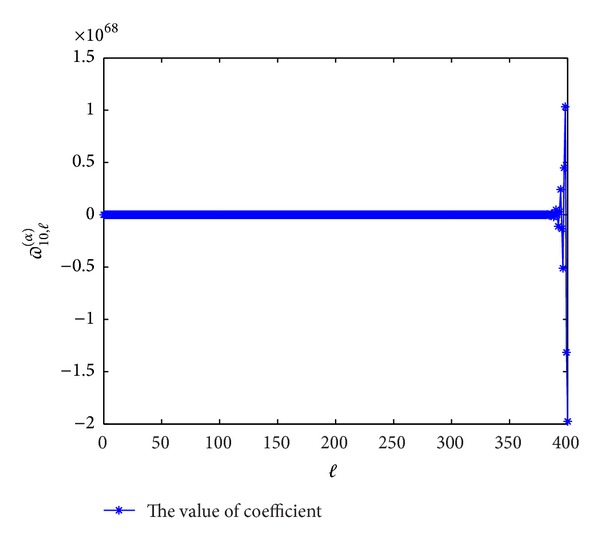
The values of the coefficients *ϖ*_10,*l*_^(*α*)^  (*l* = 0,1,…) for *α* = 0.3.

Next, we plot the figures of coefficients of *ϖ*_*p*,*l*_^(*α*)^(*p* = 7,8, 9,10) to show the evolutions with *l*. From Figures 5, 6, 7, and 8, we can see that these coefficients are violently oscillatory such that the approximation behaves like Runge's phenomenon, which is similar to the case of ordinary differential equation. So, to seek high-order (≥7th-order) schemes by this form seems not to be appropriate.

## 3. Numerical Examples

In order to verify the reasonability of the coefficients for *p* = 3,4, 5,6, we give the following two numerical examples. These numerical results show that the coefficients are efficient.

**Example 8**

Consider the function (*x*) = *x* ^*q*^(*q* = 3,4, 5,6), *x* ∈ [0,1]. The numerical absolute error and convergence order at *x* = 1 by higher-order difference scheme (14) with different *p*  (*p* = 3,4, 5,6) are shown in Tables 1, 2, 3, and 4.

**Example 9**

Let us consider a fractional ordinary differential equation

(33) DRL0,xαy(x) =Γ(8−α)Γ(8−2α)x7−2α−2880Γ(7−α)x6−α+Γ(6+α)20x5,

with initial values

(34) DRL0,xα−1y(x)|x=0=0, α∈(0,1).

The exact solution of the above equation is given by

(35) $$\begin{array}{c} y\left(x\right)=x^{7-\alpha }-4x^{6}+6x^{5+\alpha }. \end{array}$$

At this moment, we use the numerical formula (14) with different order *p* to solve this equation. The absolute error and numerical convergence order are listed in Tables 5, 6, 7, and 8.

From the numerical results presented in Tables 1–8, we can see that the coefficients of the fractional linear multistep method for *p* = 3,4, 5,6 are efficient.

The coefficients of *ϖ*_*p*,*l*_^(*α*)^  (*p* = 7,8, 9,10) are violently oscillatory which may not be suitable for numerical calculations. Now, we take an example to show this.

**Example 10**

Consider the function *f*(*x*) = *x* ^3^, *x* ∈ [0,1]. Consider

(36) DRL0,xαx3=Γ(4)Γ(4−α)x3−α.

**Figure 9:**
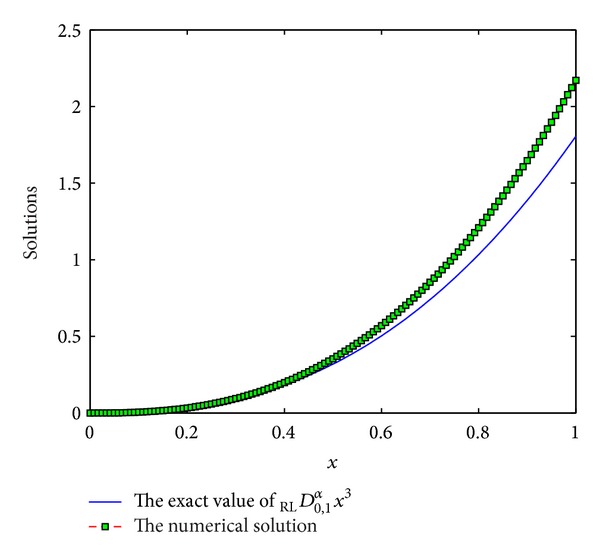
The exact value of _RL_ *D*_0,1_^*α*^ *x* ^3^ and numerical solution of *p* = 7 for *α* = 0.5 and step length of *h* = 1/120.

**Figure 10:**
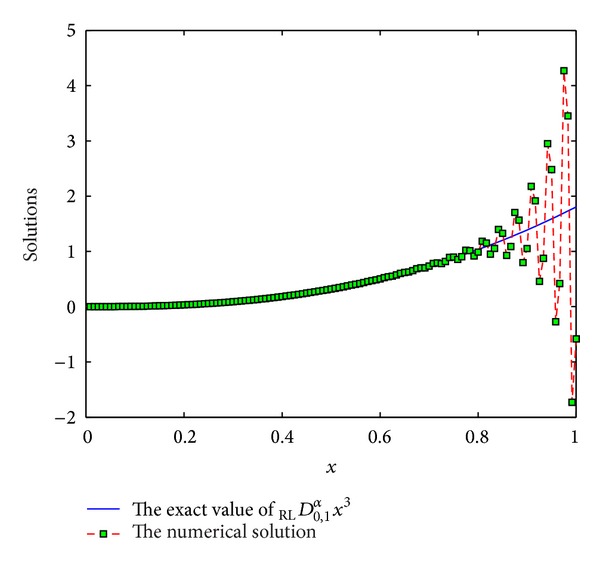
The exact value of _RL_ *D*_0,1_^*α*^ *x* ^3^ and numerical solution of *p* = 8 for *α* = 0.5 and step length of *h* = 1/120.

**Figure 11:**
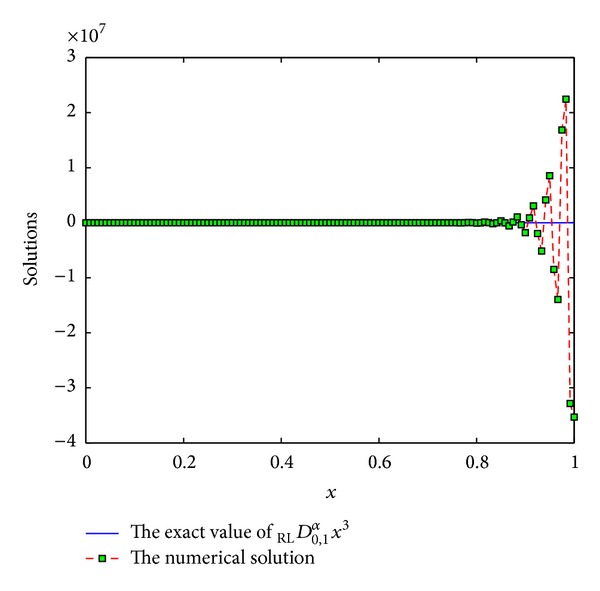
The exact value of _RL_ *D*_0,1_^*α*^ *x* ^3^ and numerical solution of *p* = 9 for *α* = 0.5 and step length of *h* = 1/120.

**Figure 12:**
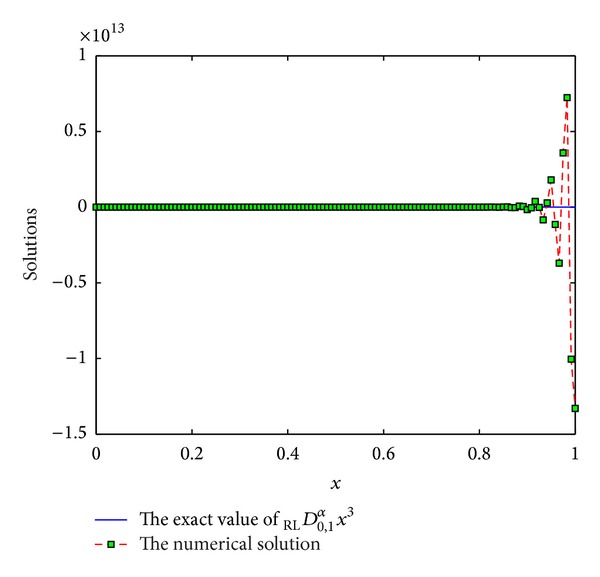
The exact value of _RL_ *D*_0,1_^*α*^ *x* ^3^ and numerical solution of *p* = 10 for *α* = 0.5 and step length of *h* = 1/120.

We use the scheme (14) with *p* = 7,8, 9,10 to numerically compute _RL_ *D*_0,*x*_^*α*^ *x* ^3^. See Figures 9, 10, 11, and 12. From these figures, we can see that the results are not numerically stable, which can be regarded as fractional Runge's phenomenon. So, it is not necessary to derive more than 6th-order schemes for Riemann-Liouville derivative by generating function method.

## 4. Conclusion

In this paper, we propose recursion formulas to compute the coefficients of the fractional linear multistep schemes. The numerical experiments have been carried out to support the derived numerical schemes. Here, we should note that the *p*th order (*p* ≥ 7) schemes are not stable.

## Figures and Tables

**Table 1:** The numerical results of Example 8 with *q* = 3 by formula (14) with *p* = 3.

*α*	*h*	The absolute error	The convergence order
0.2	1/10	2.645451*e* − 004	—
	1/20	3.262235*e* − 005	3.0196
	1/40	4.051498*e* − 006	3.0093
	1/80	5.048441*e* − 007	3.0045

0.4	1/10	4.245164*e* − 004	—
	1/20	5.164722*e* − 005	3.0391
	1/40	6.373660*e* − 006	3.0185
	1/80	7.917542*e* − 007	3.0090

0.6	1/10	4.388473*e* − 004	—
	1/20	5.266513*e* − 005	3.0588
	1/40	6.457975*e* − 006	3.0277
	1/80	7.997522*e* − 007	3.0135

0.8	1/10	2.902946*e* − 004	—
	1/20	3.435614*e* − 005	3.0789
	1/40	4.185974*e* − 006	3.0369
	1/80	5.167862*e* − 007	3.0179

**Table 2:** The numerical results of Example 8 with *q* = 4 by formula (14) with *p* = 4.

*α*	*h*	The absolute error	The convergence order
0.2	1/10	8.565957*e* − 005	—
	1/20	5.244691*e* − 006	4.0297
	1/40	3.248631*e* − 007	4.0130
	1/80	2.021606*e* − 008	4.0063

0.4	1/10	1.389875*e* − 004	—
	1/20	8.345704*e* − 006	4.0578
	1/40	5.123066*e* − 007	4.0578
	1/80	3.174360*e* − 008	4.0125

0.6	1/10	1.451794*e* − 004	—
	1/20	8.553695*e* − 006	4.0851
	1/40	5.203490*e* − 007	4.0390
	1/80	3.210299*e* − 008	4.0187

0.8	1/10	9.693830*e* − 005	—
	1/20	5.608540*e* − 006	4.1114
	1/40	3.381060*e* − 007	4.0521
	1/80	2.076976*e* − 008	4.0249

**Table 3:** The numerical results of Example 8 with *q* = 5 by formula (14) with *p* = 5.

*α*	*h*	The absolute error	The convergence order
0.2	1/5	1.243084*e* − 003	—
	1/10	3.601600*e* − 005	5.1091
	1/20	1.098356*e* − 006	5.0352
	1/40	3.392476*e* − 008	5.0169

0.4	1/5	2.128798*e* − 003	—
	1/10	5.880181*e* − 005	5.1780
	1/20	1.756897*e* − 006	5.0648
	1/40	5.363365*e* − 008	5.0337

0.6	1/5	2.304179*e* − 003	—
	1/10	6.169164*e* − 005	5.2230
	1/20	1.810078*e* − 006	5.0910
	1/40	5.461289*e* − 008	5.0507

0.8	1/5	1.548320*e* − 003	—
	1/10	4.138297*e* − 005	5.2255
	1/20	1.193106*e* − 006	5.1162
	1/40	3.557536*e* − 008	5.0677

**Table 4:** The numerical results of Example 8 with *q* = 6 by formula (14) with *p* = 6.

*α*	*h*	The absolute error	The convergence order
0.2	1/5	1.366727*e* − 003	—
	1/10	1.834110*e* − 005	6.2195
	1/20	2.831669*e* − 007	6.0173
	1/40	4.352325*e* − 009	6.0237

0.4	1/5	2.458586*e* − 003	—
	1/10	2.979151*e* − 005	6.3668
	1/20	4.561725*e* − 007	6.0292
	1/40	6.997415*e* − 009	6.0266

0.6	1/5	2.796453*e* − 003	—
	1/10	3.106344*e* − 005	6.4922
	1/20	4.745505*e* − 007	6.0325
	1/40	7.487801*e* − 009	5.9859

0.8	1/5	81.968603*e* − 003	—
	1/10	2.092182*e* − 005	6.5560
	1/20	3.168932*e* − 007	6.0449
	1/40	4.769865*e* − 009	6.0539

**Table 5:** The numerical results of Example 9 by formula (14) with *p* = 3.

*α*	*h*	The absolute error	The convergence order
0.2	1/20	8.448207*e* − 004	—
	1/40	1.101436*e* − 004	2.9393
	1/80	1.406092*e* − 005	2.9696
	1/160	1.776240*e* − 006	2.9848

0.4	1/20	2.742719*e* − 003	—
	1/40	3.594722*e* − 004	2.9317
	1/80	4.599387*e* − 005	2.9664
	1/160	5.816133*e* − 006	2.9816

0.6	1/20	6.698506*e* − 003	—
	1/40	8.826119*e* − 004	2.9240
	1/80	1.132122*e* − 004	2.9628
	1/160	1.433358*e* − 005	2.9816

0.8	1/20	1.458663*e* − 002	—
	1/40	1.931852*e* − 003	2.9166
	1/80	2.484219*e* − 004	2.9591
	1/160	3.149142*e* − 005	2.9798

**Table 6:** The numerical results of Example 9 by formula (14) with *p* = 4.

*α*	*h*	The absolute error	The convergence order
0.2	1/10	7.061963*e* − 004	—
	1/20	4.697130*e* − 005	3.9102
	1/40	3.067961*e* − 006	3.9364
	1/80	1.966873*e* − 007	3.9633

0.4	1/10	2.626111*e* − 003	—
	1/20	1.735473*e* − 004	3.9195
	1/40	1.117528*e* − 005	3.9569
	1/80	7.093827*e* − 007	3.9776

0.6	1/10	7.072031*e* − 003	—
	1/20	4.720003*e* − 004	3.9053
	1/40	3.043142*e* − 005	3.9552
	1/80	1.931056*e* − 006	3.9781

0.8	1/10	1.660047*e* − 002	—
	1/20	1.125103*e* − 003	3.8831
	1/40	7.302226*e* − 005	3.9456
	1/80	4.647896*e* − 006	3.9737

**Table 7:** The numerical results of Example 9 by formula (14) with *p* = 5.

*α*	*h*	The absolute error	The convergence order
0.2	1/5	3.255980*e* − 004	—
	1/10	2.611815*e* − 005	3.6400
	1/20	1.709675*e* − 006	3.9333
	1/40	7.187111*e* − 008	4.5722

0.4	1/5	4.502359*e* − 003	—
	1/10	1.361298*e* − 004	5.0476
	1/20	4.930325*e* − 006	4.7872
	1/40	1.689483*e* − 007	4.8670

0.6	1/5	1.726870*e* − 002	—
	1/10	4.858555*e* − 004	5.1515
	1/20	1.493401*e* − 005	5.0239
	1/40	4.654620*e* − 007	5.0038

0.8	1/5	4.809699*e* − 002	—
	1/10	1.421046*e* − 003	5.0809
	1/20	4.352885*e* − 005	5.0288
	1/40	1.348082*e* − 006	5.0130

**Table 8:** The numerical results of Example 9 by formula (14) with *p* = 6.

*α*	*h*	The absolute error	The convergence order
0.2	1/5	1.624059*e* − 003	—
	1/10	1.004444*e* − 005	7.3371
	1/20	4.333263*e* − 007	4.5348
	1/40	7.431670*e* − 009	5.8656

0.4	1/5	3.378083*e* − 003	—
	1/10	1.682553*e* − 006	10.9713
	1/20	3.570178*e* − 007	2.2366
	1/40	7.098342*e* − 009	5.6524

0.6	1/5	4.899244*e* − 003	—
	1/10	1.910223*e* − 005	8.0027
	1/20	3.435689*e* − 008	9.1189
	1/40	2.811996*e* − 009	3.6109

0.8	1/5	4.824629*e* − 003	—
	1/10	3.070796*e* − 005	7.2957
	1/20	3.129379*e* − 007	6.6166
	1/40	3.973945*e* − 009	6.2992

## Appendix

### The Computations of Coefficients *ϖ*_*p*,*l*_^(*α*)^ for Case *p* = 3,4, 5,6

Here, we first introduce the Fourier transform method to compute the coefficients for *p* = 3,4, 5,6. Letting *z* = *e* ^*iθ*^ and substituting it into (15) yield

(A.1) $$\begin{array}{c} W_{p}^{\left(\alpha \right)}\left(e^{i\theta }\right)=\overset{\infty }{\underset{l=0}{\sum }}\varpi_{p,l}^{\left(\alpha \right)}e^{il\theta },\;p=2,3,4,5,6. \end{array}$$

So, the coefficients *ϖ*_*p*,*l*_^(*α*)^ can be expressed as

(A.2) $$\begin{array}{c} \varpi_{p,l}^{\left(\alpha \right)}=\frac{1}{2\pi }\overset{2\pi }{\underset{0}{\int }}W_{p}^{\left(\alpha \right)}\left(e^{i\theta }\right)e^{-il\theta }d\theta ,\;p=2,3,4,5,6. \end{array}$$

That is, we need compute the following integrals:

(A.3) ϖ2,l(α)=12π∫02πW2(α)(eiθ)e−ilθdθ=12π∫02π(32−2eiθ+12ei2θ)αe−ilθdθ,ϖ3,l(α)=12π∫02πW3(α)(eiθ)e−ilθdθ=12π∫02π(116−3eiθ+32ei2θ−13ei3θ)αe−ilθdθ,ϖ4,l(α)=12π∫02πW4(α)(eiθ)e−ilθdθ=12π∫02π(2512−4eiθ+3ei2θ−43ei3θ+14ei4θ)α ×e−ilθdθ,ϖ5,l(α)=12π∫02πW5(α)(eiθ)e−ilθdθ=12π∫02π(13760−5eiθ+5ei2θ−103ei3θ+54ei4θ−15ei5θ)αe−ilθdθ,ϖ6,l(α)=12π∫02πW6(α)(eiθ)e−ilθdθ=12π∫02π(14760−6eiθ+152ei2θ−203ei3θ+154ei4θ−65ei5θ+16ei6θ)αe−ilθdθ.

Obviously, it is not an easy task to obtain the explicit analytical expressions of the coefficients *ϖ*_*p*,*l*_^(*α*)^  (*p* = 2,3, 4,5, 6) if *α* ∉ *Z*. These analytical expressions are seemingly complicated but are very useful for theoretical analysis, such as stability and convergence analysis. In the following, we give an effective and interesting method to obtain the explicit analytical expressions of coefficients *ϖ*_*p*,*l*_^(*α*)^  (*p* = 2,3, 4,5, 6). For case *p* = 2, it is easy to get; see [27] for more details,

(A.4) $$\begin{array}{c} \varpi_{2,l}^{\left(\alpha \right)}={\left(\frac{3}{2}\right)}^{\alpha }\overset{l}{\underset{l_{1}=0}{\sum }}{\left(\frac{1}{3}\right)}^{l_{1}}\varpi_{1,l_{1}}^{\left(\alpha \right)}\varpi_{1,l-l_{1}}^{\left(\alpha \right)},\;l=0,1,\ldots . \end{array}$$

For cases *p* = 3,4, 5,6, the details are given as follows.

(i) Consider *p* = 3.

By some calculations, one has

(A.5) W3(α)(z)=(116−3z+32z2−13z3)α=(116)α(1−z)α(1−711z+211z2)α=(116)α(1−z)α∑l1=0∞(αl1)(−711z)l1(1−27z)l1=(116)α(1−z)α∑l1=0∞(αl1)(−711z)l1∑l2=0l1(l1l2)(−27z)l2=(116)α(1−z)α∑l1=0∞[∑l2=0[(1/2)l1](αl1−l2)(−711)l1−l2(l1−l2l2)(−27)l2]zl1=(116)α[∑l1=0∞(αl1)(−z)l1]×{∑l1=0∞[∑l2=0[(1/2)l1](αl1−l2)(−711)l1−l2×(l1−l2l2)(−27)l2]zl1}=(116)α∑l=0∞[∑l1=0l∑l2=0[(1/2)l1](−1)l(711)l1−l2(27)l2(αl−l1)(αl1−l2)(l1−l2l2)]zl=(116)α∑l=0∞[∑l1=0l∑l2=0[(1/2)l1](−1)l2(711)l1−l2(27)l2(l1−l2)!l2!(l1−2l2)!G1,l−l1(α)G1,l1−l2(α)]zl.

Comparing the above equation and (14) with *p* = 3 gives

(A.6) ϖ3,l(α)=(116)α∑l1=0l∑l2=0[(1/2)l1](−1)l2(711)l1−l2(27)l2×(l1−l2)!l2!(l1−2l2)!ϖ1,l−l1(α)ϖ1,l1−l2(α),l=0,1,….

(ii) Consider *p* = 4.

By little tedious calculations, one gets

(A.7) W4(α)(z)=(2512−4z+3z2−43z3+14z4)α=(2512)α(1−z)α(1−2325z+1325z2−325z3)α=(2512)α(1−z)α∑l1=0∞(αl1)(−2325z)l1(1−1323z+323z2)l1=(2512)α(1−z)α∑l1=0∞(αl1)(−2325z)l1∑l2=0l1(l1l2)(−1323z)l2(1−313z)l2=(2512)α(1−z)α∑l1=0∞(αl1)(−2325z)l1∑l2=0l1(l1l2)(−1323z)l2∑l3=0l2(l2l3)(−313z)l3=(2512)α(1−z)α∑l1=0∞(αl1)(−2325z)l1 ×∑l2=02l1[∑l3=max⁡⁡{0,l2−l1}[(1/2)l2](l1l2−l3)(−1323)l2−l3(l2−l3l3)(−313)l3]zl2=(2512)α(1−z)α∑l1=0∞[∑l2=0[(2/3)l1]∑l3=max⁡{0,2l2−l1}[(1/2)l2](αl1−l2)(−2325)l1−l2×(l1−l2l2−l3)×(−1323)l2−l3(l2−l3l3)(−313)l3]zl1=(2512)α[∑l1=0∞(αl1)(−z)l1] ×{∑l1=0∞[∑l2=0[(2/3)l1]∑l3=max⁡{0,2l2−l1}[(1/2)l2](αl1−l2)×(−2325)l1−l2(l1−l2l2−l3)×(−1323)l2−l3(l2−l3l3)(−313)l3]zl1}=(2512)α∑l=0∞[∑l1=0l∑l2=0[(2/3)l1]∑l3=max⁡{0,2l2−l1}[(1/2)l2](−1)l(2325)l1−l2(1323)l2−l3(313)l3×(αl−l1)(αl1−l2)×(l1−l2l2−l3)(l2−l3l3)‍‍]zl=(2512)α∑l=0∞[∑l1=0l∑l2=0[(2/3)l1]∑l3=max⁡⁡{0,2l2−l1}[(1/2)l2](−1)l2(2325)l1−l2(1323)l2−l3(313)l3×(l1−l2)!l3!(l2−2l3)!(l1+l3−2l2)!×ϖ1,l−l1(α)G1,l1−l2(α)]zl

It follows from the above equation and (14) with *p* = 4 that

(A.8) ϖ4,l(α)=(2512)α∑l1=0l∑l2=0[(2/3)l1]∑l3=max⁡{0,2l2−l1}[(1/2)l2](−1)l2(2325)l1−l2(1323)l2−l3(313)l3‍×(l1−l2)!l3!(l2−2l3)!(l1+l3−2l2)!ϖ1,l−l1(α)ϖ1,l1−l2(α),l=0,1,….

(iii) Consider *p* = 5.

By complex computations, one obtains

(A.9) W5(α)(z)=(13760−5z+5z2−103z3+54z4−15z5)α=(13760)α(1−z)α(1−163137z+z2−63137z3+12137z4)α=(13760)α(1−z)α∑l1=0∞(αl1)(−163137z)l1(1−137163z+63163z2−12163z3)l1=(13760)α(1−z)α∑l1=0∞(αl1)(−163137z)l1∑l2=0l1(l1l2)(−137163z)l2×(1−63137z+12137z2)l2=(13760)α(1−z)α∑l1=0∞(αl1)(−163137z)l1∑l2=0l1(l1l2)(−137163z)l2 ×∑l3=0l2(l2l3)(−63137z)l3∑l4=0l3(l3l4)(−421z)l4=(13760)α(1−z)α∑l1=0∞(αl1)(−163137z)l1∑l2=0l1(l1l2)(−137163z)l2 ×∑l3=02l2[∑l4=max⁡{0,l3−l2}[(1/2)l3](l2l3−l4)(−63137)l3−l4(l3−l4l4)(−421)l4]zl3=(13760)α(1−z)α∑l1=0∞(αl1)(−163137z)l1 ×∑l2=03l1[∑l3=max⁡⁡{0,l2−l1}[(2/3)l2] ∑l4=max⁡⁡{0,2l3−l2}[(1/2)l3](−137163)l2−l3(−63137)l3−l4(−421)l4×(l1l2−l3)(l2−l3l3−l4)(l3−l4l4)]zl2=(13760)α(1−z)α ×∑l1=0∞[∑l2=0[(3/4)l1]∑l3=max⁡⁡{0,2l2−l1}[(2/3)l2] ∑l4=max⁡⁡{0,2l3−l2}[(1/2)l3](−163137)l1−l2(−137163)l2−l3(−63137)l3−l4×(−421)l4(αl1−l2)×(l2−l3l3−l4)(l3−l4l4)]zl1=(13760)α[∑l1=0∞(αl1)(−z)l1] ×{∑l1=0∞∑l2=0[(3/4)l1]∑l3=max⁡⁡{0,2l2−l1}[(2/3)l2] ∑l4=max⁡⁡{0,2l3−l2}[(1/2)l3](−1)l1(163137)l1−l2(137163)l2−l3(63137)l3−l4×(421)l4(αl1−l2)(l1−l2l2−l3)×(l2−l3l3−l4)(l3−l4l4)zl1}=(13760)α∑l=0∞[∑l1=0l∑l2=0[(3/4)l1]∑l3=max⁡⁡{0,2l2−l1}[(2/3)l2]‍×∑l4=max⁡⁡{0,2l3−l2}[(1/2)l3](−1)l(163137)l1−l2(137163)l2−l3(63137)l3−l4(421)l4×(αl−l1)(αl1−l2)(l1−l2l2−l3)×(l2−l3l3−l4)(l3−l4l4)]zl=(13760)α∑l=0∞[∑l1=0l∑l2=0[(3/4)l1]∑l3=max⁡⁡{0,2l2−l1}[(2/3)l2]‍×∑l4=max⁡⁡{0,2l3−l2}[(1/2)l3](−1)l2(163137)l1−l2(137163)l2−l3(63137)l3−l4(421)l4×(l1−l2)!l4!(l3−2l4)!(l1+l3−2l2)!(l2+l4−2l3)!×ϖ1,l−l1(α)G1,l1−l2(α)]zl

So we have

(A.10) ϖ5,l(α)=(13760)α∑l1=0l∑l2=0[(3/4)l1]∑l3=max⁡⁡{0,2l2−l1}[(2/3)l2]  ×∑l4=max⁡⁡{0,2l3−l2}[(1/2)l3](−1)l2(163137)l1−l2(137163)l2−l3(63137)l3−l4(421)l4×(l1−l2)!l4!(l3−2l4)!(l1+l3−2l2)!(l2+l4−2l3)!ϖ1,l−l1(α)ϖ1,l1−l2(α),l=0,1,….

(iv) Consider *p* = 6.

By tedious calculations, one has

(A.11) W6(α)(z)=(14760−6z+152z2−203z3+154z4−65z5+16z6)α=(14760)α(1−z)α(1−213147z+237147z2−163147z3+62147z4−10147z5)α=(14760)α(1−z)α∑l1=0∞(αl1)(−213147z)l1(1−237213z+163213z2−62213z3+10213z4)l1=(14760)α(1−z)α∑l1=0∞(αl1)(−213147z)l1∑l2=0l1(l1l2)(−237213z)l2×(1−163237z+62237z2−10237z3)l2=(14760)α(1−z)α∑l1=0∞(αl1)(−213147z)l1∑l2=0l1(l1l2)(−237213z)l2 ×∑l3=0l2(l2l3)(−163237z)l3(1−62163z+10163z2)l3=(14760)α(1−z)α∑l1=0∞(αl1)(−213147z)l1∑l2=0l1(l1l2)(−237213z)l2×∑l3=0l2(l2l3)(−163237z)l3 ×∑l4=0l3(l3l4)(−62163z)l4(1−531z)l4=(14760)α(1−z)α∑l1=0∞(αl1)(−213147z)l1∑l2=0l1(l1l2)(−237213z)l2×∑l3=0l2(l2l3)(−163237z)l3 ×∑l4=0l3(l3l4)(−62163z)l4∑l5=0l4(l4l5)(−531z)l5=(14760)α(1−z)α∑l1=0∞(αl1)(−213147z)l1∑l2=0l1(l1l2)(−237213z)l2×∑l3=0l2(l2l3)(−163237z)l3 ×∑l4=02l3[∑l5=max⁡⁡{0,l4−l3}[(1/2)l4](−62163)l4−l5(−531)l5×(l3l4−l5)(l4−l5l5)]zl4=(14760)α(1−z)α∑l1=0∞(αl1)(−213147z)l1∑l2=0l1(l1l2)(−237213z)l2 ×∑l3=03l2[∑l4=max⁡{0,l3−l2}[(2/3)l3] ∑l5=max⁡{0,2l4−l3}[(1/2)l4](−163237)l3−l4(−62163)l4−l5‍×(−531)l5×(l2l3−l4)(l3−l4l4−l5)(l4−l5l5)]zl3=(14760)α(1−z)α∑l1=0∞(αl1)(−213147z)l1 ×∑l2=04l1[∑l3=max⁡⁡{0,l2−l1}[(3/4)l2] ∑l4=max⁡⁡{0,2l3−l2}[(2/3)l3]×∑l5=max⁡⁡{0,2l4−l3}[(1/2)l4](−237213)l2−l3(−163237)l3−l4(−62163)l4−l5(−531)l5×(l1l2−l3)(l2−l3l3−l4)(l3−l4l4−l5)(l4−l5l5)]zl2=(14760)α(1−z)α ×∑l1=0∞[∑l2=0[(4/5)l1]∑l3=max⁡⁡{0,2l2−l1}[(3/4)l2] ∑l4=max⁡⁡{0,2l3−l2}[(2/3)l3] ×∑l5=max⁡⁡{0,2l4−l3}[(1/2)l4](−213147)l1−l2(−237213)l2−l3(−163237)l3−l4×(−62163)l4−l5(−531)l5×(αl1−l2)(l1−l2l2−l3)(l2−l3l3−l4)×(l3−l4l4−l5)(l4−l5l5)]zl1=(14760)α[∑l1=0∞(αl1)(−z)l1] ×{∑l1=0∞[∑l2=0[(4/5)l1]∑l3=max⁡⁡{0,2l2−l1}[(3/4)l2] ∑l4=max⁡⁡{0,2l3−l2}[(2/3)l3] ×∑l5=max⁡⁡{0,2l4−l3}[(1/2)l4](−213147)l1−l2(−237213)l2−l3×(−163237)l3−l4(−62163)l4−l5×(−531)l5(αl1−l2)×(l1−l2l2−l3)(l2−l3l3−l4)×(l3−l4l4−l5)(l4−l5l5)]zl1}=(14760)α ×∑l=0∞[∑l1=0l∑l2=0[(4/5)l1]∑l3=max⁡⁡{0,2l2−l1}[(3/4)l2] ∑l4=max⁡⁡{0,2l3−l2}[(2/3)l3] ×∑l5=max⁡⁡{0,2l4−l3}[(1/2)l4](−1)l−l1(−213147)l1−l2(−237213)l2−l3(−163237)l3−l4×(−62163)l4−l5(−531)l5×(αl−l1)(αl1−l2)(l1−l2l2−l3)×(l2−l3l3−l4)(l3−l4l4−l5)(l4−l5l5)]zl=(14760)α ×∑l=0∞[∑l1=0l∑l2=0[(4/5)l1]∑l3=max⁡⁡{0,2l2−l1}[(3/4)l2] ∑l4=max⁡⁡{0,2l3−l2}[(2/3)l3]×∑l5=max⁡⁡{0,2l4−l3}[(1/2)l4](−1)l2(237213)l2−l3(163237)l3−l4(62163)l4−l5(531)l5×(l1−l2)!l5!(l4−2l5)!(l1+l3−2l2)!(l2+l4−2l3)!(l3+l5−2l4)!×ϖ1,l−l1(α)ϖ1,l1−l2(α)]zl.

Hence, we can obtain the sixth-order coefficients as follows:

(A.12) ϖ6,l(α)=(14760)α ×∑l1=0l∑l2=0[(4/5)l1]∑l3=max⁡{0,2l2−l1}[(3/4)l2] ∑l4=max⁡{0,2l3−l2}[(2/3)l3]  ×∑l5=max⁡{0,2l4−l3}[(1/2)l4](−1)l2(213147)l1−l2(237213)l2−l3×(163237)l3−l4(62163)l4−l5(531)l5×(l1−l2)!l5!(l4−2l5)!(l1+l3−2l2)!(l2+l4−2l3)!(l3+l5−2l4)!×ϖ1,l−l1(α)ϖ1,l1−l2(α), l=0,1,….

The main reason to list the exact expression of the coefficients is that they are helpful for stability and convergence analysis. The coefficients for *p* = 7,8, 9,10 are omitted due to inefficiency.
